# Adaptive strategies based on shrub leaf-stem anatomy and their environmental interpretations in the eastern Qaidam Basin

**DOI:** 10.1186/s12870-024-05026-3

**Published:** 2024-04-24

**Authors:** Siyu Liu, Jingming Zheng

**Affiliations:** Beijing Key Laboratory of Forest Resource Ecosystem Processes, Beijing Forestry University, Beijing, 100083 China

**Keywords:** Eastern Qaidam Basin, Shrub, Leaves anatomical traits, Stems anatomical traits, Adaptive strategies, Water stress

## Abstract

**Background:**

Water stress seriously affects the survival of plants in natural ecosystems. Plant resistance to water stress relies on adaptive strategies, which are mainly based on plant anatomy with following relevant functions: (1) increase in water uptake and storage; (2) reduction of water loss; and (3) mechanical reinforcement of tissues. We measured 15 leaf-stem anatomical traits of five dominant shrub species from 12 community plots in the eastern Qaidam Basin to explore adaptive strategies based on plant leaf-stem anatomy at species and community levels. and their relationship with environmental stresses were tested.

**Results:**

Results showed that the combination of leaf-stem anatomical traits formed three types of adaptive strategies with the drought tolerance of leaf and stem taken as two coordinate axes. Three types of water stress were caused by environmental factors in the eastern Qaidam Basin, and the established adaptive strategy triangle could be well explained by these environmental stresses. The interpretation of the strategic triangle was as follows: (1) exploitative plant strategy, in which leaf and stem adopt the hydraulic efficiency strategy and safety strategy, respectively. This strategy is mostly applied to plants in sandy desert (i.e., *Nitraria tangutorum*, and *Artemisia sphaerocephala*) which is mainly influenced by drought stress; (2) stable plant strategy, in which both leaf/assimilation branches and stem adopt hydraulic safety strategy. This strategy is mostly applied to plants in salty desert (i.e., *Kalidium foliatum* and *Haloxylon ammodendron*) which aridity has little effect on them; and (3) opportunistic plant strategy, in which leaf and stem adopt hydraulic safety strategy and water transport efficiency strategy. This strategy is mostly applied to plants in multiple habitats (i.e., *Sympegma regelii*) which is mainly affected by coldness stress.

**Conclusion:**

The proposed adaptive strategy system could provide a basis for elucidating the ecological adaptation mechanism of desert woody plants and the scientific management of natural vegetation in the Qinghai-Tibet Plateau.

**Supplementary Information:**

The online version contains supplementary material available at 10.1186/s12870-024-05026-3.

Many parts of the world are constrained by water scarcity as a result of global climate change. The Qaidam Basin is located in the northeast of the Qinghai-Tibet Plateau and is a typical desert area. The eastern part of the Qaidam Basin is an area with relatively more precipitation and rich biodiversity, which is of great value for the study of desert plants. Water stress affects the survival of plants in natural ecosystems, and adverse environmental conditions such as drought, freezing, and high soil salinity may lead plants to facing water stress [1]. Plants have different structures and anatomy due to differences in their sensitivity and adaptive response to water stress. As all biogeochemical processes are driven by climate, increasing water stress could impact the Carbon-gains and Carbon-losses of ecosystems as well as plant growth by affecting its phenology and structural modifications at biochemical, physiological, and anatomical levels (e.g., leaf shedding reduces transpiration and water loss, and leaf thickening improves water retention capacity) [2–4]. Among them, the anatomical structure is the most direct manifestation of plants in response to water stress, which has greater research value. Understanding the response of plants to increasing water stress could help in forecasting dynamics in natural ecosystems and in adjusting management practices in agriculture under global climate change [4].

Every plant organ is ideally designed to fulfill metabolic and physiological processes in specific environmental conditions [4]. In environments characterized by aridity, plant survival depends on the ability to integrate its structure and function to withstand desiccation without permanent damage [5]. The structural variances in these plants, which we call the adaptive strategies of plants, are mainly related to water saving (e.g., water storage and reduction of water losses) and mechanical reinforcement of tissues (e.g., thickening of cell walls) [6–8]. Anatomical traits are hard traits and are intuitive characteristics expressed by adapting to comprehensive environmental conditions, which are closely related to plant functions and adaptive strategies [9]. Different from physiological, biochemical, and other indicators, all morpho-anatomical attributes are interpreted under the perspective that not a single trait but suites of anatomical features are responsible through certain synergy and trade-offs for the adaptive capacity of plants in a specific environment [4]. When two or more anatomical traits are consistently correlated among species, they may be considered to form a strategic axis (or spectrum) of trait variation, thus forming a plant’s adaptive strategy [10–14].

Among aboveground organs of desert plants, leaves are the main organs for plant photosynthesis and transpiration, which play a vital role in plant growth and development [15–17]. Stems have the main functions of water transport and mechanical support, in addition to functions of storage and reproduction [18]. Stems and leaves play an important role in the process of plant adaptation to water stress, and their anatomical characteristics can be used as proxies for plant morphological variances and a series of physiological and biochemical processes [19, 20]. Most studies revealed that reduced stomata and increased leaf thickness can improve drought resistance ability of leaves by improving water storage and reducing water loss [4]. Stem anatomical traits adapt to the environment through trade-offs between xylem water transport efficiency and hydraulic safety [4].

Transpiration can be reduced through reduction of leaf number and size, leaf shedding, etc. In addition to morphological appearance, resistance to water stress can be characterized by the anatomical features at internal and surface of the leaf [8]. Variations of leaf anatomy with habitats suggest that plants can adapt to environmental changes by adjusting the proportions of leaf tissues [19, 20]. Previous studies indicated that leaf epidermis and mesophyll thickness show significant geographic patterns at the species and community levels [21–23]. Thickening of the leaves, mesophyll, and epidermis might limit gas exchange but reduce water loss and increase water retention [8, 24, 25]. Water losses are also greatly affected by stomata characteristics, and stomata density is more plastic in response to water stress than stomata size. For example, an experimental study demonstrated that stomata density increases under moderate water deficit, whereas it declines under severe drought [26]. Under water stress conditions, changes in leaf morphology and anatomical properties can increase water storage and reduce transpiration and water loss, which makes the leaves shifting from high hydraulic efficiency to hydraulic safety, thereby improving the drought tolerance of leaves [4].

The anatomical traits of the stem can be interpreted based on their functions in species adaptive strategies. They determine the hydraulic conductivity (efficiency) and vulnerability to cavitation (safety) of given stem xylem [27–33]. The vessel characteristics are directly related to the utilization of water by the stems and are the most direct features to infer stem adaptive strategies. For example, a comparative study of 328 *Compositae* species revealed that vessel size decreases and vessel density increase during the progression from moderate habitats through dry to desert habitats [34]. For woody angiosperms, wood anatomy and wood density determined hydraulic conductivity and mechanical strength [33]. In addition, high cortical thickness and pith proportion contributed to the improvement of stem safety, and high xylem proportion increased water transport area in stem [35]. Adaptation to drought can be achieved through a sort of compromise between the need to maintain high water conductivity when water is available and to prevent embolism under conditions of aridity. For many xylem anatomical features, there was existence of direct or indirect proportionality with hydraulic efficiency/safety can be traced. Such relations are sometimes ambiguous, considering that different combinations of various characteristics can tip the balance toward one extreme or the other [4]. However, a certain degree of either-or relationship undoubtedly exists between the two strategies. This balance between water transport efficiency and safety is well achieved by many shrub species in Mediterranean semiarid ecosystems, whose stems have high conductivity when water is abundant (low vessel density, larger vessel size) while keep safe during drought periods (high vessel density, smaller vessel size) [36, 37].

The adaptive strategies of plants formed by their morphological and anatomical features have been well defined in recent studies. Leaf anatomical traits adapt to water stress, change from hydraulic efficiency to hydraulic safety strategy, while stem anatomical traits adapt to water stress with trade-offs between water transport efficiency and safety strategies [4]. Plant adaptation to water stress is neither controlled by a single anatomical trait nor is it determined by a single organ [4]. The association between the adaptative strategies of stems and leaves is similar to population features in the CSR (competitor, stress-tolerator, ruderal theory) life history strategies [38] (Fig. 1). CSR life history countermeasures are an important theory of plant function strategy proposed by Grime, which divides the life history of plants based on the intensity and severity of habitat disturbance [38]. Obviously, no combination of low leaf hydraulic efficiency and low stem water transport efficiency exists in desert plants, the leaf-stem anatomical structure in desert plants will likely form an adaptive strategy system similar to Grime’s CSR life history strategies. So far, adaptive strategy of desert plants was rarely studied by anatomical traits, and such adaptive strategies system defined by the association between stem and leaf traits is unknown. We hypothesized that the combination of stem and leaf adaptative strategies based on their anatomical traits for shrubs in the Qaidam Basin can be defined and used to explain the species distribution and the response of species to water stress in this special environment.

**Fig. 1:**
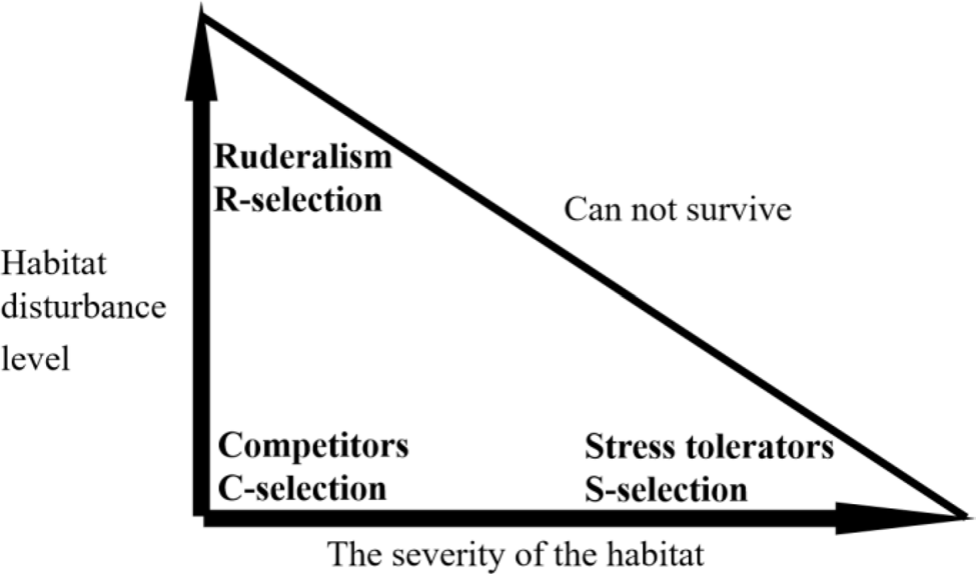
Grime’s CSR life history strategy triangle

In this study, we sampled the dominant shrubs in the eastern Qaidam Basin and measured their stem and leaf anatomical traits. We also tried to explore the adaptive strategies of stem and leaf using anatomical traits of desert shrubs at both species and community levels. Conditions of water stress in the eastern Qaidam Basin were clarified by combining a variety of environmental indicators, and their effects on shrub stem and leaf adaptive strategies were explored. On this basis, an adaptive strategy system of desert shrubs based on stem and leaf anatomical traits was established by defining the adaptation of stem and leaf anatomy to drought as two coordinate axes. By synthesized the results of species and community adaptive strategies, species morphological structure and environmental stress characteristics were jointly verified.

## Materials and methods

### Study sites

**Fig. 2:**
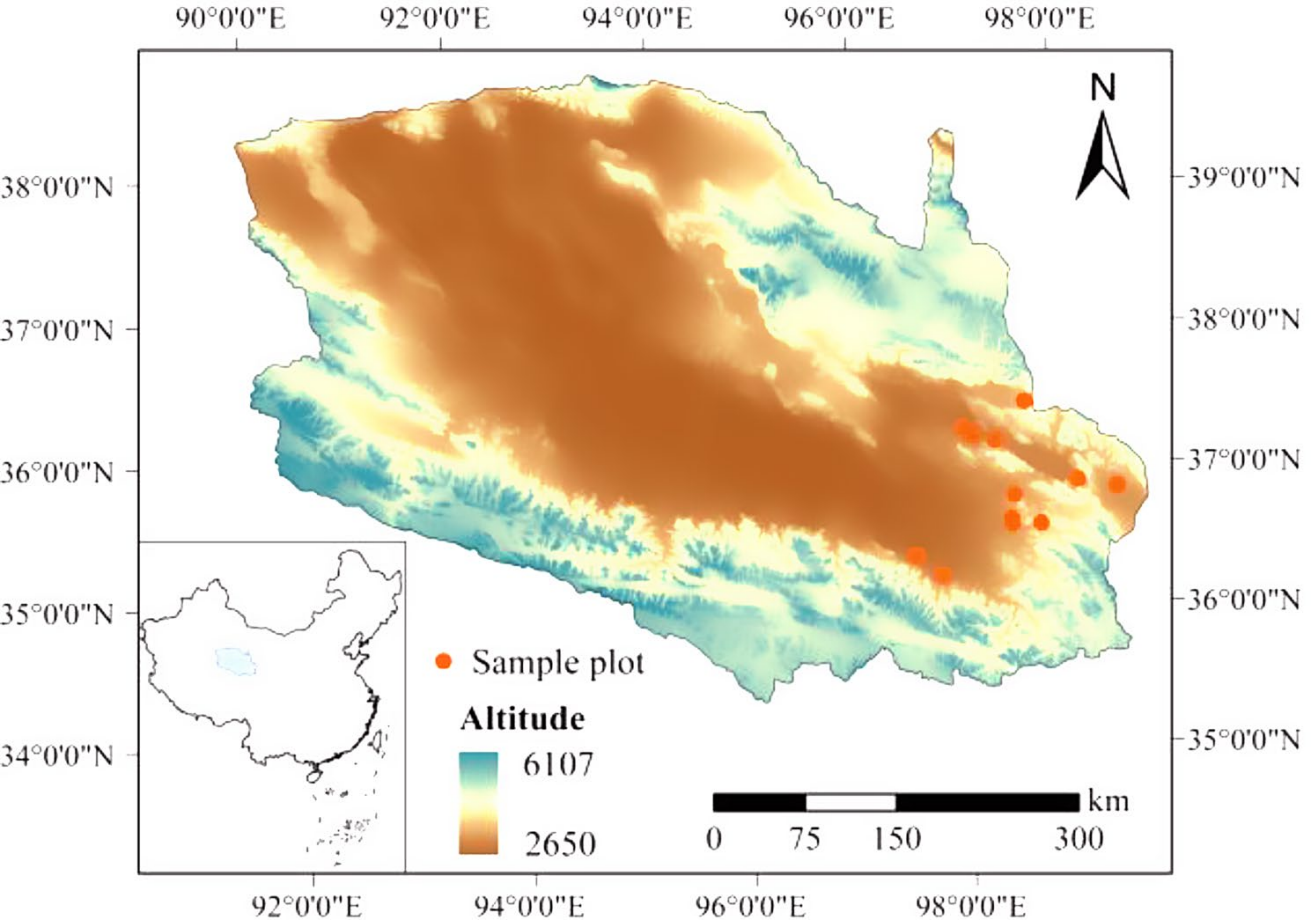
Location of the Qaidam Basin and sampling sites (The map is open sourced from [44])

The Qaidam Basin is a large enclosed intermountain fault basin and is one of the four major basins in China. It is located in the northwest of Qinghai Province and the northeast of the Qinghai-Tibet Plateau, mainly in the Haixi Mongol-Tibetan Autonomous Prefecture [39]. The study area is located in the eastern part of the Qaidam Basin, a latitude between 36°03′ N and 37°21′ N and a longitude between 97°11′ E and 99°01′ E. and the altitude is between 2857 and 3555 m, spanning Delingha City, Dulan County, Ulan County, etc. (Fig. 2). The study area has an arid temperate continental climate with annual precipitation much less than evaporation [40]. The climate is dry and windy in spring. In summer, precipitation is more frequent and concentrated and the climate is relatively humid thus suitable for the growth of cold-tolerant and drought-resistant plant species. The temperature during autumn cools down sharply, and climate in winter is dry and cold [41]. In Qaidam basin, the mean annual relative humidity is 30–40%, and the minimum can be less than 5%. The mean annual temperature is below 5 °C with an absolute annual temperature difference more than 60 °C, a daily temperature difference around 30 °C, and temperature during summer nights can drop below 0 °C [42]. The number of windy days above grade 8 can reach 25–75 days per year, strong winds of 40 m/s can even occur, and the wind erosion is serious in this region.

The Qaidam Basin is composed of various desert lands. The main soil types are saline desert soil and gypsum desert soil, and soil salinization is obvious. The vegetation in the study area is sparse consisting of a few plant species with a total number less than 80, mainly shrubs, semi-shrubs, and herbs with high drought resistance, and many halophytes are present. The vegetation structure is simple, and more than half of the flora are composed of one or several species. From August 2020, 12 plots surveyed in the eastern Qaidam Basin were selected for community-level trait analysis (Table S1). Since the aboveground biomass of five dominant species in the 12 plots was greater than 85% of the community biomass (Table 1, species information is shown in Table S2), community weighted mean (CWMs) of species traits within the plot were used to represent the trait characteristics at the plot level [43].

**Table 1:** Species information and biomass share in the plot

Site	Dominant species	Relative abundance (%)	Biomass proportion (%)
**P01**	*Sympegma regelii*	58.802	
	*Haloxylon ammodendron*	38.518	97.320
**P02**	*Sympegma regelii*	76.545	
	*Artemisia sphaerocephala*	18.869	95.414
**P03**	*Nitraria tangutorum*	99.502	99.502
**P04**	*Artemisia sphaerocephala*	93.930	93.930
**P05**	*Kalidium foliatum*	100.000	100.000
**P06**	*Kalidium foliatum*	85.421	85.421
**P07**	*Nitraria tangutorum*	25.693	
	*Artemisia sphaerocephala*	58.891	
	*Haloxylon ammodendron*	15.415	100.000
**P08**	*Nitraria tangutorum*	62.597	
	*Haloxylon ammodendron*	33.769	96.366
**P09**	*Kalidium foliatum*	100.000	100.000
**P10**	*Kalidium foliatum*	88.720	88.720
**P11**	*Sympegma regelii*	100.000	100.000
**P12**	*Kalidium foliatum*	98.228	98.228

### Sample collection and anatomical trait measurement

A total of 15 functional traits were selected in this study including seven leaf anatomical traits, six stem anatomical traits, and two commonly used economic traits (Table 2). The leaf anatomical traits are total leaf area (LA), single leaf area (La), leaf thickness (LT), epidermal thickness (ET), mesophyll thickness (MT), stomata density (SD), and stomata length (SL). The stem anatomical traits include stem sapwood area (SA), cortical thickness (CT), xylem area to sapwood area (XS), pith area to sapwood area (PS), vessel density (VD), and vessel size (VS). We also selected leaf mass per area (LMA) and wood density (WD). LMA is an important leaf carbon economic trait and has a significant impact on leaf photosynthetic capacity [45]. LMA represents the proportion of carbon investment in leaves, which is significantly related to the protection ability of leaves [45]. WD is an important carbon economic trait of stems and a key trait indicating tradeoff between stem water transport efficiency and hydraulic safety [46]. WD represents the proportion of carbon investment in the stem, which is significantly correlated with the mechanical strength of the stem [46]. Specific data are shown in Table S2−4.

Three representative individuals of the dominant species in each plot were selected, and 3–4 healthy branches of nearly the same size in the middle or upper plant canopy were collected by pruning shears for each plant. The whole branch of the sample was removed, and all the leaves were spread to avoid overlay. The sample was flattened, placed on standard mesh paper in the Scanner (LIDE 200, China). Clear images scanned were used for LA and La measurement. After scanning, 4–5 well-grown leaves were selected on the branch sample as leaf anatomy samples, and 2–3 cm long branch was cut off at branch bottom as stem anatomy samples. All anatomical samples were fixed and stored with FAA fixative solution (100 mL FAA = 90 mL 70% ethanol + 5 mL 37% formaldehyde + 5 mL 99.5% glacial acetic acid) immediately after removal and brought back to the laboratory [47]. A plant slicer (MTH−1, DL Nature Gene Life Science, Inc., Japan) was used to obtain the leaf cross section and stem cross section with a thickness of 10–20 μm by making temporary tissue slides [48]. The slides were observed and photographed under a light microscope (*N*−117 M, Ningbo Yongxin Optical Co., Ltd., China), and the surface stomatal features of leaf samples were observed and photographed by scanning electron microscopy (SEM, S−3400 N, Hitachi, Japan). All image measurements were performed using ImageJ software (National Institutes of Health, NIH, U.S.) [49].

The scanned plant branches were sealed in envelopes and brought back to the laboratory for drying in a 60 °C oven for 72 h. After being dried, the leaves were weighed to obtain the leaf dry weight, i.e., ratio of leaf dry weight to leaf area. Wood density was determined by Achimedes method [50]. Fresh stem samples were immersed in water to measure their volume, then naturally air-dried and weighted using a balance with 0.0001 accuracy for wood density.

The community weighted mean (CWM) of each trait is calculated by the following formula:

1 $$CWM = \sum\limits_{i = 1}^{\text{n}} {Pi \times trai{t_i}\quad i = 1,2,3 \ldots n}$$

where *Pi* is the relative biomass of species *i* in the community, *n* is the total number of species in the community, and *trait* is the trait value of species *i* [51].

**Table 2:** Abbreviations and description for the traits in the text

Acronym	Leaf and stem traits	Description
LMA	Leaf mass per area (g/cm²)	The ratio of specific leaf weight to total leaf area.
LA	Total leaf area (cm^2^)	The sum of all leaf areas on the entire branches.
La	Single leaf area (cm^2^)	Average of the upper leaf area of the entire branches.
LT	Leaf thickness (cm)	The thickness of the leaf cross-section.
ET	Epidermal thickness (µm)	The thickness of the outermost part of the leaf.
MT	Mesophyll thickness (µm)	The thickness between the upper and lower epidermis, include the palisade tissue and the sponge tissue (Psammophyte sponge tissue is specialized as a water-storing tissue composed of large parenchyma cells [52–54]).
SD	Stomata density (mm-²)	The number of stomata per unit area.
SL	Stomata length (µm)	Average of the maximum length of the stomata.
WD	Wood density (g/ml)	The ratio of stem segment mass and volume.
SA	Sapwood area (cm2)	The cross-sectional area at the very bottom of the branches.
CT	Cortical thickness (µm)	The thickness of the outermost part of the mature stem.
XS	Xylem area to sapwood area (%)	The ratio of stem xylem area to stem cross-sectional area.
PS	Pith area to sapwood area (%)	The ratio of stem pith area to stem cross-sectional area.
VD	Vessel density (mm-²)	The number of vessels per unit area.
VS	Vessel size (µm)	Average of the maximum length of the vessel.

### Environmental data

There are 19 environmental factors for vegetation plots including nine climatic indicators, four drought indices, and 6 soil indicators (Table 3). Climate indicators and drought indices were obtained from raster data provided by the National Earth System Science Data Center [44], including mean annual precipitation (MAP), mean precipitation during growing season (MPS), average precipitation in August (AP8), mean annual evaporation (MAE), mean annual humidity (MAH), mean annual air temperature (MAT), mean temperature during growing season (MTS), average temperature in August (AT8), mean annual wind speed (MAW), standardized precipitation index (SPI), standardized precipitation evapotranspiration index (SPEI), Palmer drought index (PDSI), and aridity Index (AI).

Soil indicators included pH of soil (pH), soil soluble salt concentration (EC), water content of soil (WS), soil hardness (SH), soil bulk density (BD), and soil organic matter content (SOM). Soil samples were taken and measured in the field with three random soil pits stratified into seven depths within each plot (0–5, 5–10, 10–20, 20–40, 40–60, 60–80, 80–100 cm). The soil samples obtained were divided into two batches, one batch was immediately weighed to obtain the fresh weight and placed in zip lock bags, then brought to laboratory for drying. After soil dry weight was obtained, WS was calculated, and BD was determined by the cutting ring method. Another batch of samples was brought back to the laboratory and naturally air-dried for all physical and chemical index experiments. The pH of the soil was measured by applying the potentiometry method [55]. Soil EC was measured by electrode method. Soil hardness was determined on site using a soil-hardness meter (HJSD-J−750-I, Yueqing Hemu Instrument Co., Ltd., China) [56]. SOM was determined by dichromate oxidation [57]. Specific environmental data are provided in Table S5.

**Table 3:** Parameters and abbreviations for environmental indicators in the text

Indicator type	Acronym	Environmental indicators	Unit
Drought indices	SPI	Standardized precipitation index	unitless
Drought indices	SPEI	Standardized precipitation evapotranspiration index	unitless
Drought indices	PDSI	Palmer drought index	unitless
Drought indices	AI	Aridity Index	unitless
Soil indicator	pH	pH of soil	unitless
Soil indicator	EC	Soil soluble salt concentration	ms/cm
Soil indicator	WS	Water content of soil	%
Soil indicator	SH	Soil hardness	Pa
Soil indicator	BD	Soil bulk density	g/cm3
Soil indicator	SOM	Soil organic matter	g/kg
Climatic indicator	MAP	Mean annual precipitation	mm
Climatic indicator	MPS	Mean precipitation during growing season	mm
Climatic indicator	AP8	Average precipitation at August	mm
Climatic indicator	MAE	Mean annual evaporation	mm
Climatic indicator	MAH	Mean annual humidity	%
Climatic indicator	MAT	Mean annual air temperature	℃
Climatic indicator	MTS	Mean temperature during growing season	℃
Climatic indicator	AT8	Average temperature at August	℃
Climatic indicator	MAW	Mean annual wind speed	m/s

### Statistical analysis

Principal component analysis (PCA) was performed on stem and leaf anatomical traits at species and community levels to clarify plant adaptive strategies. The first three axes of PCA were used for analysis. Thus, the positions of species and plots could be drawn in a three-dimensional way. After standardizing these PCs values, we used ternary diagram to express positions of species and plots in the three PCs space.

Redundancy analysis (RDA) was performed using 19 environmental factors and 15 trait indicators to determine the explanatory rate and main control factors of environmental indicators. The correlation analysis between the four drought indices and 15 other environmental factors was used to clarify the changes in soil and climate indicators with the change of drought severity. The correlation analysis between environmental factors and anatomical traits with high contribution rate of the top three axes of PCA was carried out to explore the influence of environment on adaptive strategies and adaptive variation of anatomical traits.Redundancy analysis (RDA) was performed using 19 environmental factors and 15 trait indicators to determine the explanatory rate and main control factors of environmental indicators. The correlation analysis between the four drought indices and 15 other environmental factors was used to clarify the changes in soil and climate indicators with the change of drought severity. The correlation analysis between environmental factors and anatomical traits with high contribution rate of the top three axes of PCA was carried out to explore the influence of environment on adaptive strategies and adaptive variation of anatomical traits.

PCA was performed using the ggbiplot package [58] and factoextra package [59] of R 4.2.1 (GNU General Public License, version 2) [60]. Correlation analysis was performed using the psych package [61] and ggplot2 package [62]. RDA was drawn using Canoco5 (5.15, Cabit Information Technology Co., Ltd., China) [63]. Ternary diagrams were drawn using Origin (2023b, OriginLab, Inc., U.S.) [39].

## Results

### Plant adaptive strategies summarized by leaf-stem anatomical traits at species and community level

**Fig. 3:**
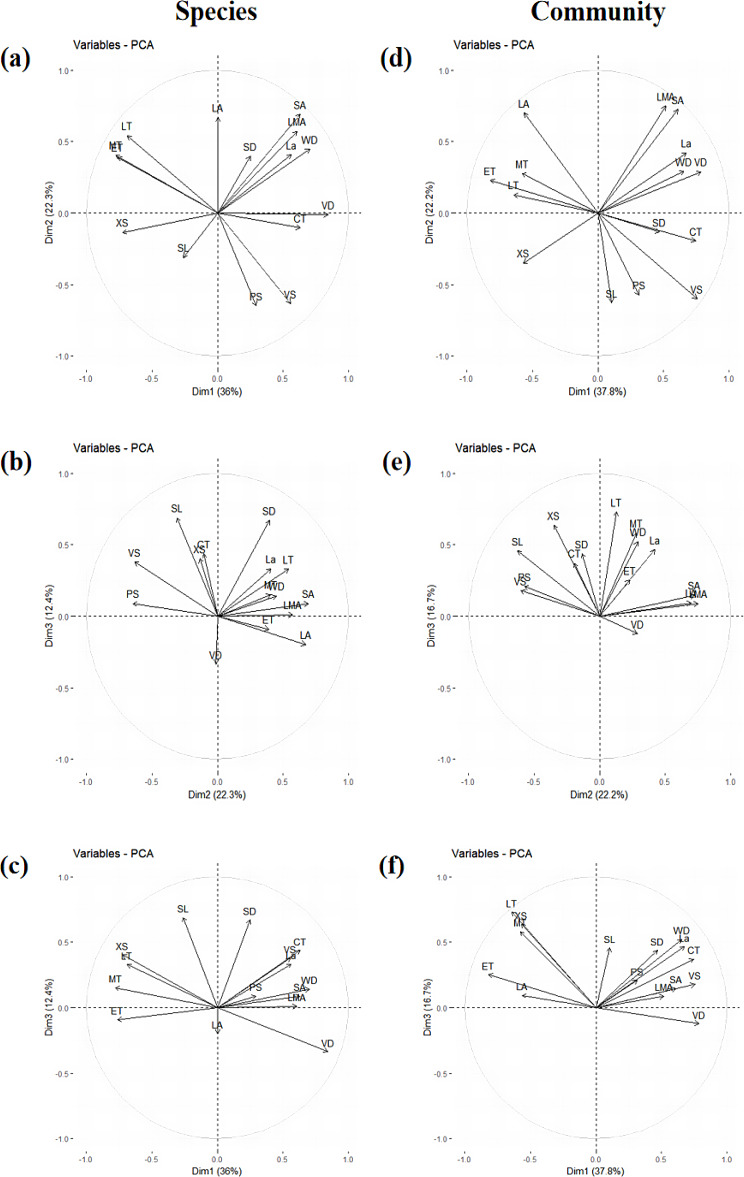
PCA of anatomical traits at species level (**a**-**c**) and community level (**d**-**f**). The images in the first column (**a**-**c**) show the species-level PCA results. The images in the second column (**d**-**f**) show the results of PCA at the community level. Images (**a**, **d**) in the first row show the results of the first and second axes. Images (**b**, **e**) in the second row are the results of the second and third axes. Images (**c**, **f**) in the third row show the results of the first and third axes

**Fig. 4:**
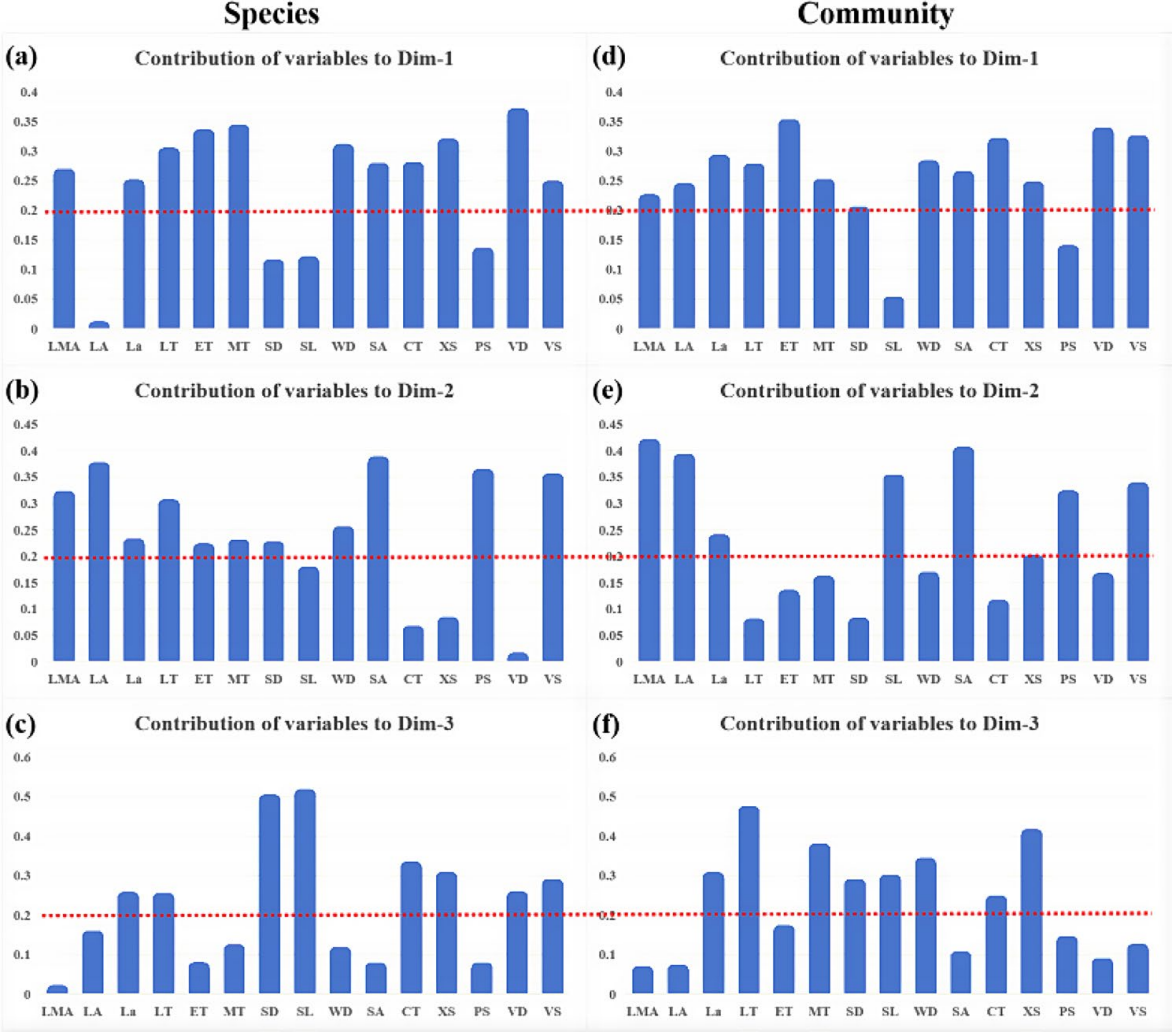
Histogram of the interpretation rate of traits at the species level (**a**-**c**) and community level (**d**-**f**) on each axis. The pictures in the first column (**a**-**c**) show the interpretation rate of each axis trait at the species level. The pictures in the second column (**d**-**f**) show the interpretation rate of each axis trait at the community level. Pictures (**a**, **d**) in the first row are the results of the first axis. The images (**b**, **e**) in the second row are the results of the second axis. Pictures (**c**, **f**) in the third row are the results of the third axis. The value of the red dotted line is 0.20, and more than 0.20 indicates that the contribution rate of the trait is high

The PCA results at species level (Figs. 3 and 4, Table S7−8 species) showed that explanation rate for the first three PCs is 70.8%. PC1 explained 36.0% of the total variance. The anatomical traits with high PC1 explanation rate included LMA, La, LT, ET and MT for leaf and WD, SA, CT, XS, VD and VS for stem. PC1 was mainly related to leaf water use efficiency and stem safety. The PC2 explanation rate was 22.3%. Anatomical traits with higher explanation rates on PC2 included the LMA, LA, La, LT, ET, MT and SD for leaf and the WD, SA, PS, and VS for stem. PC2 was mainly related to the hydraulic safety of leaves and the safety for stem. The PC3 explanation rate was 12.4%, and the anatomical traits with higher PC3 explanation rate were the La, LT, SD, and SL for leaf and the CT, XS, VD, and VS for stem. PC3 was mainly related to hydraulic safety of leaves and water transport efficiency in stems xylem.

The PCA results of community traits showed that explanation rate for the first three PCs is 76.7%. PC1 explanation rate was 37.8%, and the anatomical traits with higher PC1 explanation rates included the LMA, LA, La, LT, ET, MT and SD of leaves and the WD, SA, CT, XS, VD, and VS of stems (Figs. 3 and 4, Table S7−8 community). The PC2 explanation rate was 22.2%, and the anatomical traits with higher PC2 explanation rate included the LMA, LA, La and SL of leaves, and the SA, PS, and VS of stems. The PC3 explanation rate was 16.7%, and the anatomical traits with higher PC3 explanation rates included the La, LT, MT, SD and SL of leaves, and the WD, CT, and XS of stems. The functional interpretation of the first three axes in community trait PCA was similar to those in species trait PCA. Combined with the results of the first three axes of PCA at the species and community levels, the traits with high contribution rates on the first major axis were LMA, La, LT, ET, MT of leaves and WD, SA, CT, XS, VD and VS of stems. The traits with higher contribution rates on the second axis were LMA, LA and La of leaves and SA, PS and VS of stems. The traits with higher contribution rates on the third main axis were La, LT, SD and SL of leaves and CT and XS of stems. The dimensionality reduction results of the first three spindles were shown in Table S9.

**Fig. 5:**
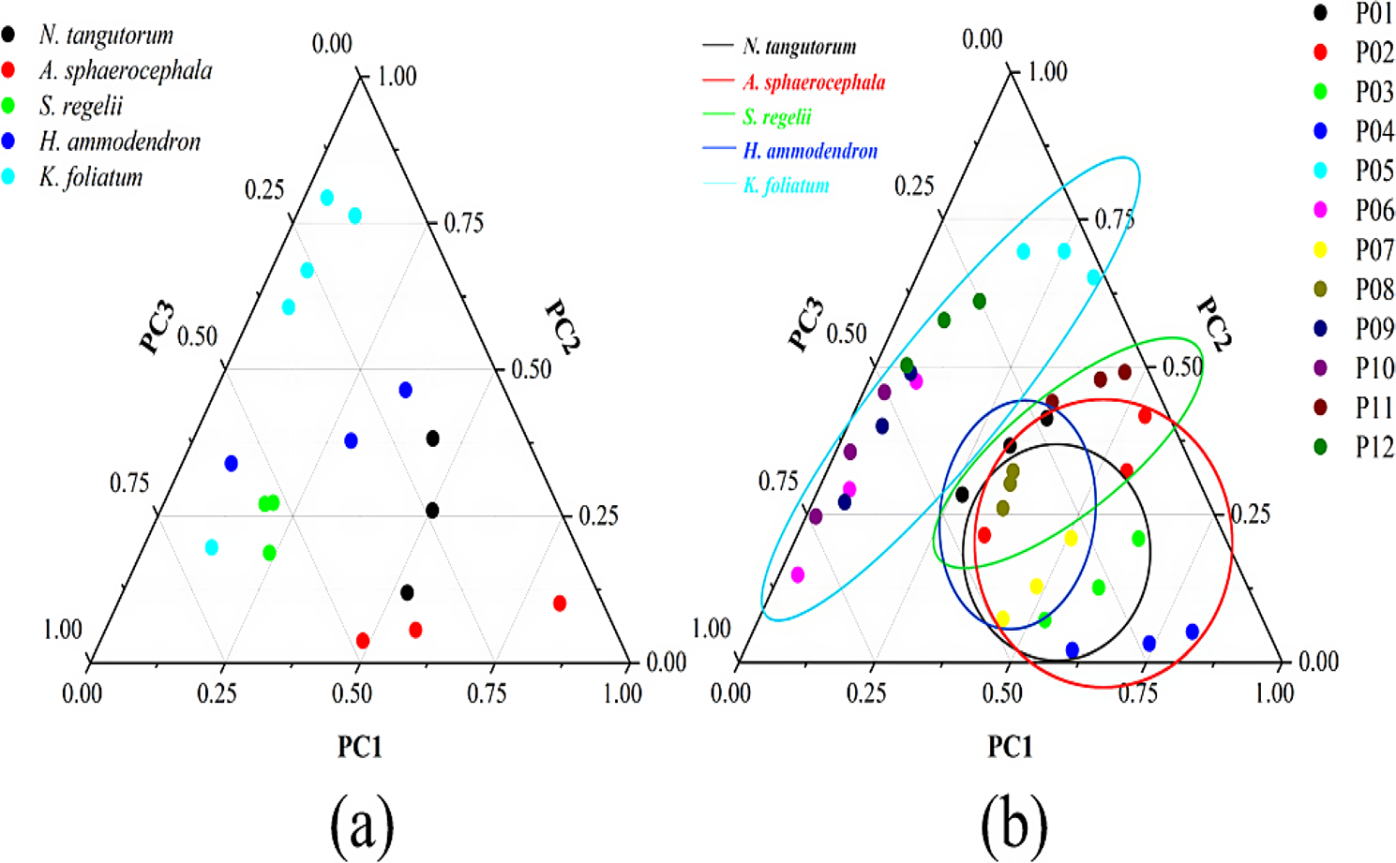
Distribution of the species (**a**) and plots (**b**) in the adaptive strategy space

In the ternary diagram of species and communities (Fig. 5), we found that the positions of *N. tangutorum* and *A. sphaerocephala* trended to the first main axis, the positions of *K. foliatum* and *H. ammodendron* trended to the second main axis, and the position of *S. regelii* trended to the third main axis. Meanwhile, the positions of *K. foliatum* and *H. ammodendron* also was a little near the third main axis. In the positions of community plots, we circled the distribution of plots by the positions of dominant species and found that the results were similar to the distribution results of species.

### Relationship between leaf-stem adaptive strategies and environmental stresses

**Fig. 6:**
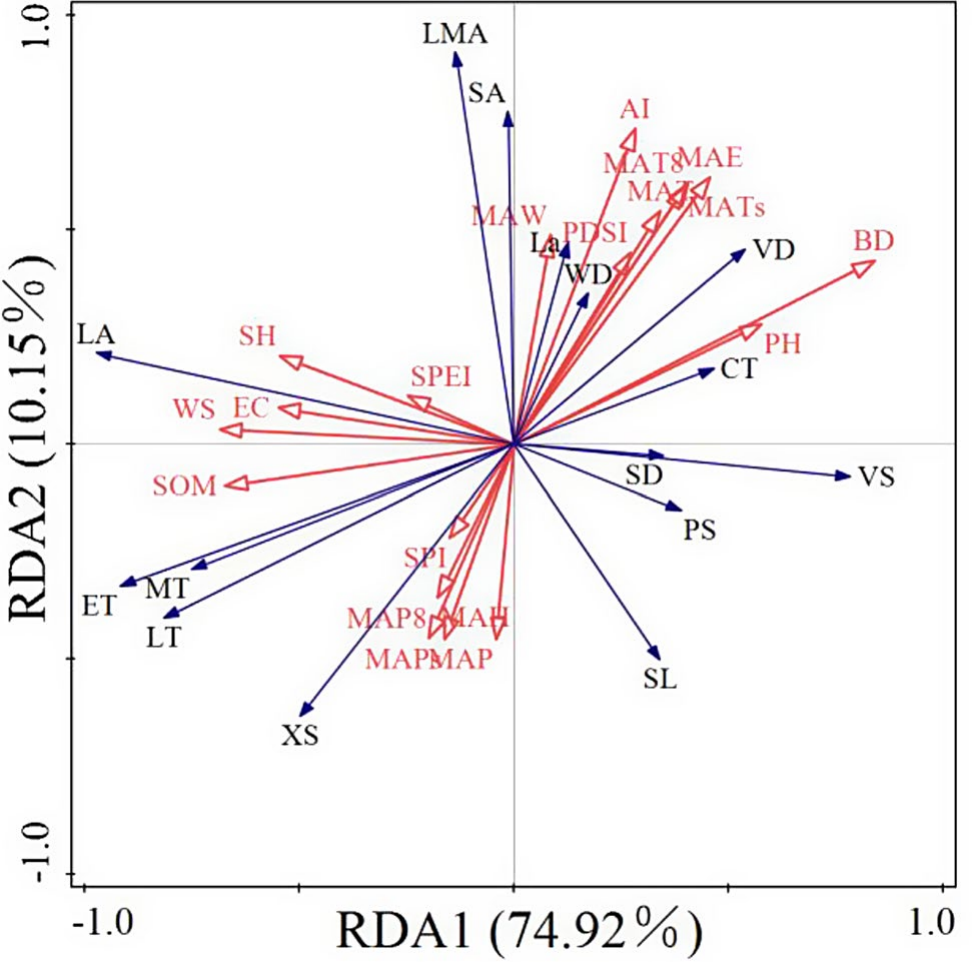
Redundancy analysis (RDA) of environmental factors and traits. The red arrows represent environmental factors, and the blue arrows represent trait indicators

The results of redundancy analysis (RDA) between environment and traits (Fig. 6) showed that the interpretation rate of the first two axes was 85.07%, and the interpretation rate of the first axis was 74.92%. The 19 environmental factors have a high explanatory rate for the eastern Qaidam Basin. Most of the environmental factors with a higher contribution rate on the first axis were soil indicators, and AI had a higher explanatory rate on the second axes in the drought index, while other drought indices did not have a high explanatory rate.

**Fig. 7:**
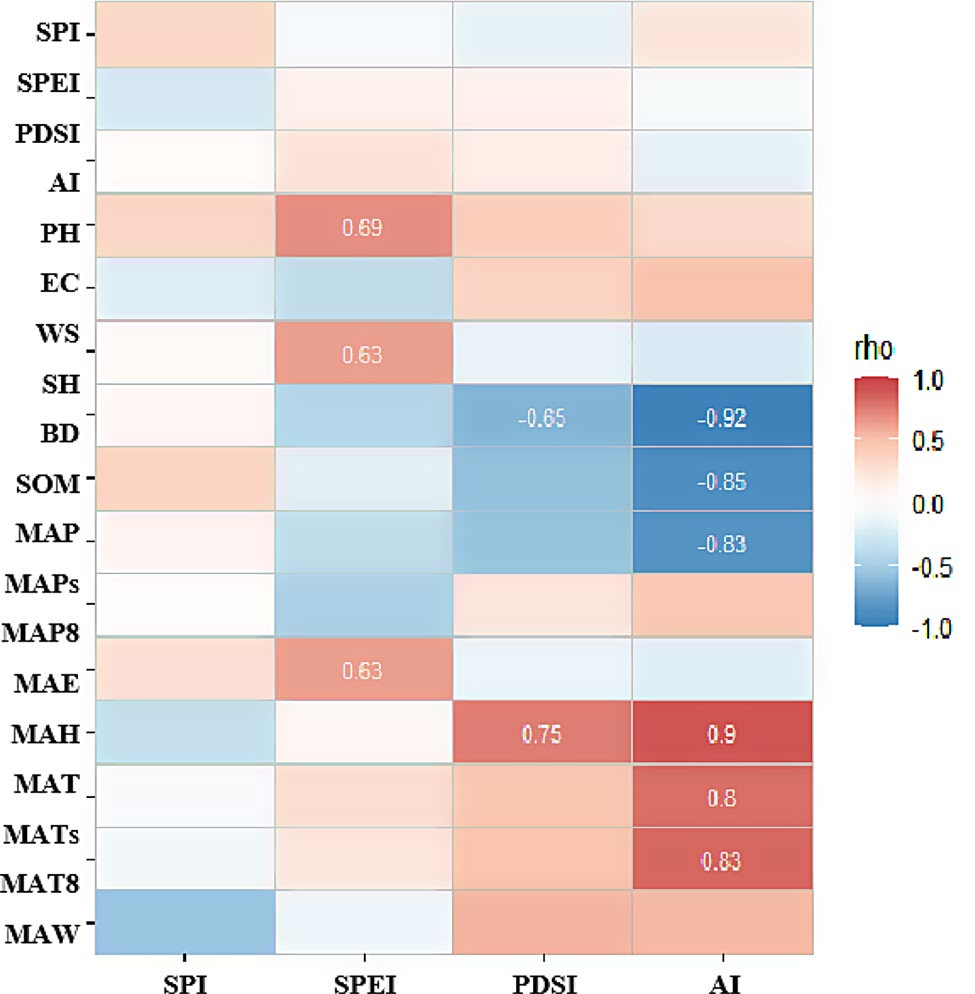
Correlation analysis of drought index to other environmental indicators

In the drought index, AI had the highest correlation with environmental factors, and SPI had a low correlation with environmental factors. The results of correlation analysis showed that as the drought intensity increased (the drought index decreases), the precipitation increased and the temperature decreased (Fig. 7).

**Fig. 8:**
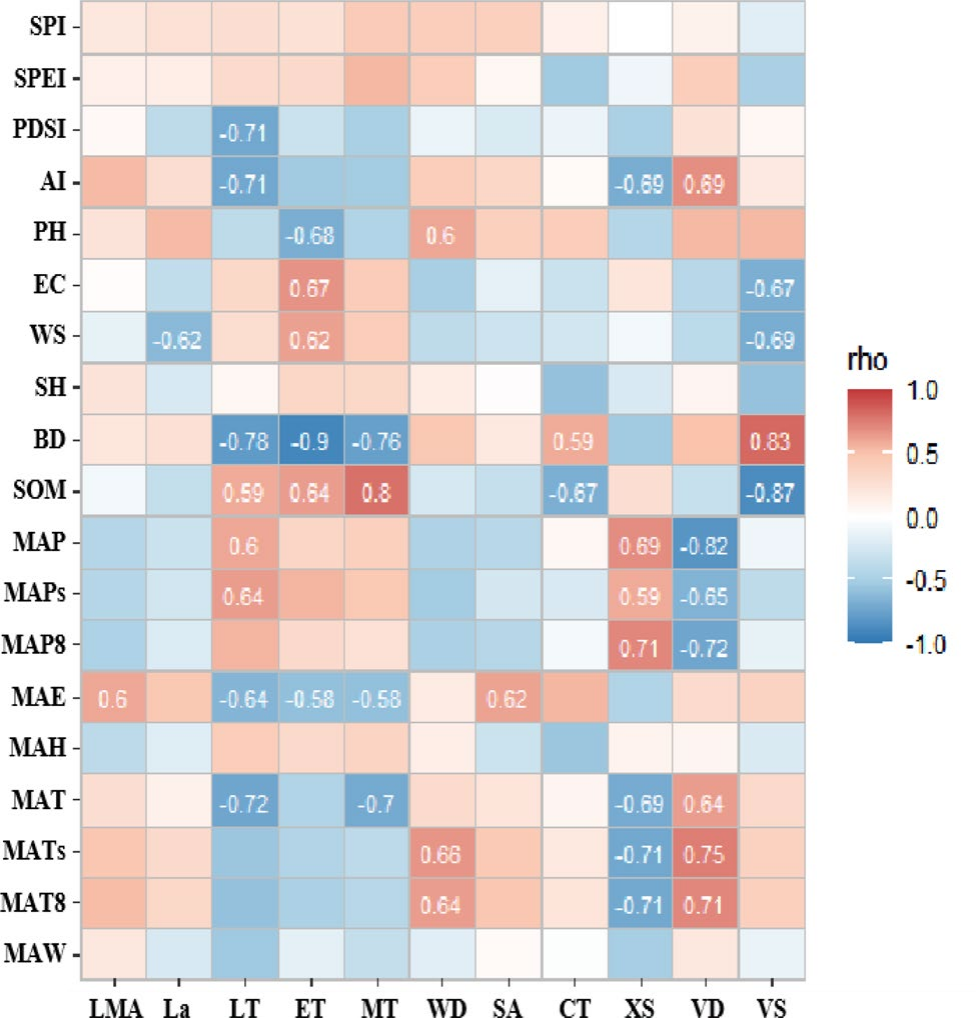
Correlation analysis between high contribution rate traits of PCA first spindle and environmental factors

The correlation analysis results between the high contribution rate traits of the first major axis of PCA and environmental factors (Fig. 8) showed that the adaptability of LT and ET of leaves and XS and VD of stems was higher in environmental variability. The variation of leaf anatomical traits was more sensitive to soil indexes, and stem anatomical traits were more sensitive to climatic indicators.

**Fig. 9:**
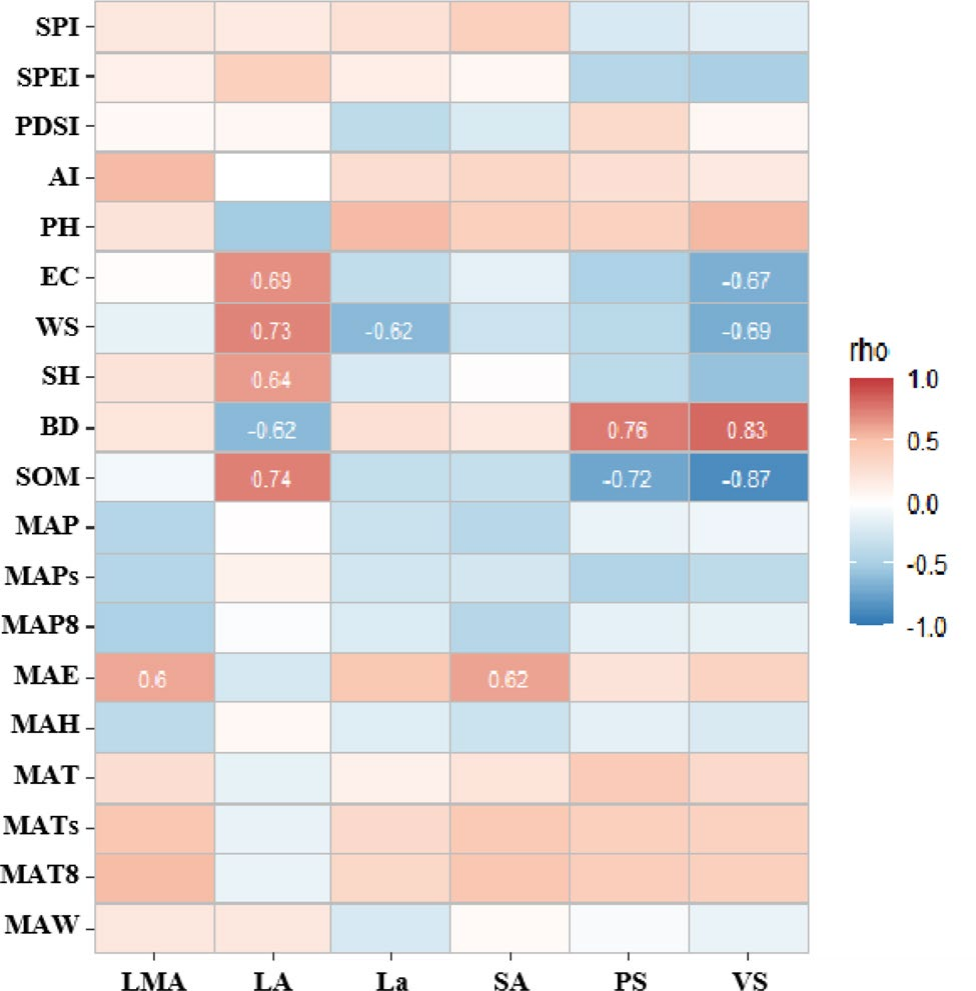
Correlation analysis between high contribution rate traits of PCA second spindle and environmental factors

The results of correlation analysis between the high contribution rate traits of the second axis of PCA and environmental factors (Fig. 9) showed that the adaptability of LA of leaves and PS and XS of stems to the environment was higher. The anatomical traits of the axial stem and leaf were basically more sensitive to soil indexes.

**Fig. 10:**
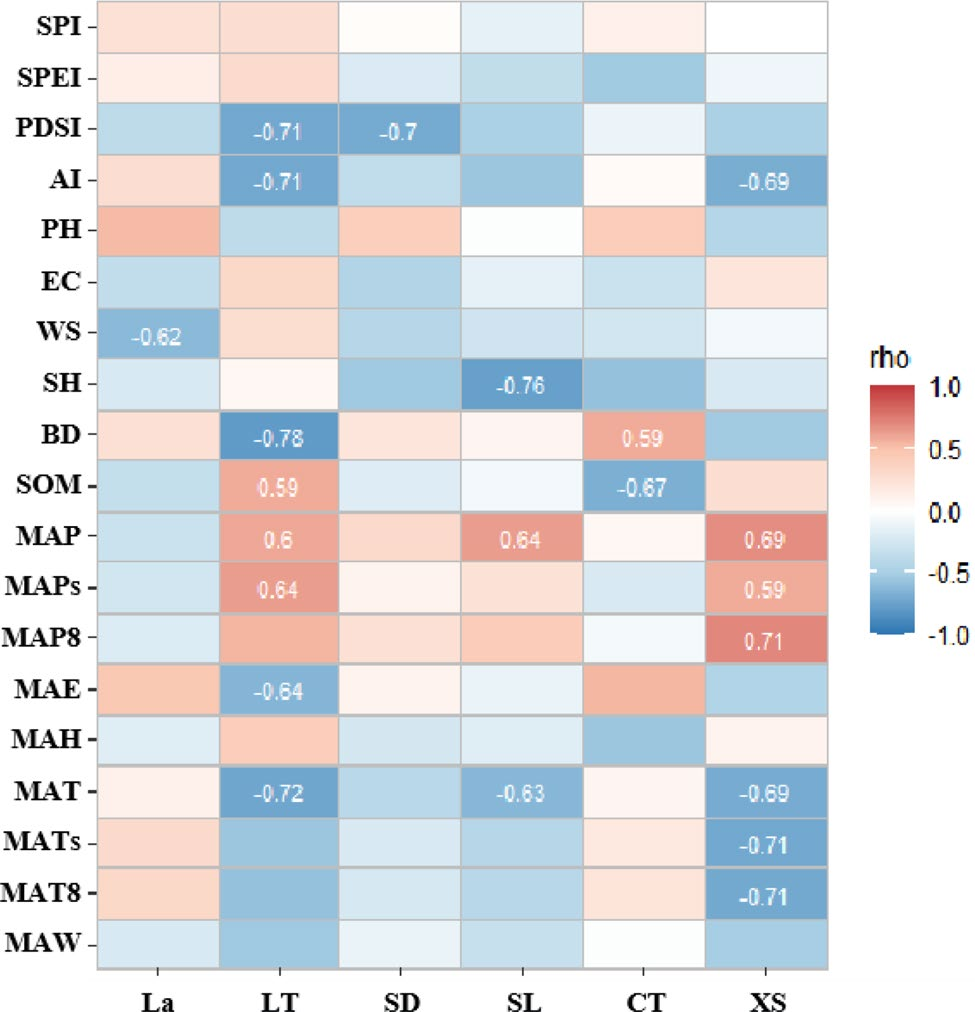
Correlation analysis between high contribution rate traits of PCA third spindle and environmental factors

The correlation analysis results between the high contribution rate traits of the third axis of PCA and environmental factors (Fig. 10) showed that the adaptability of leaves LT and stems XS to the environment were higher. LT and CT had a high correlation with soil BD and SOM, and LT and XS had a high correlation with climate indicators.

## Discussion

### A new adaptive strategy system based on anatomical traits of shrub stem and leaf

**Fig. 11:**
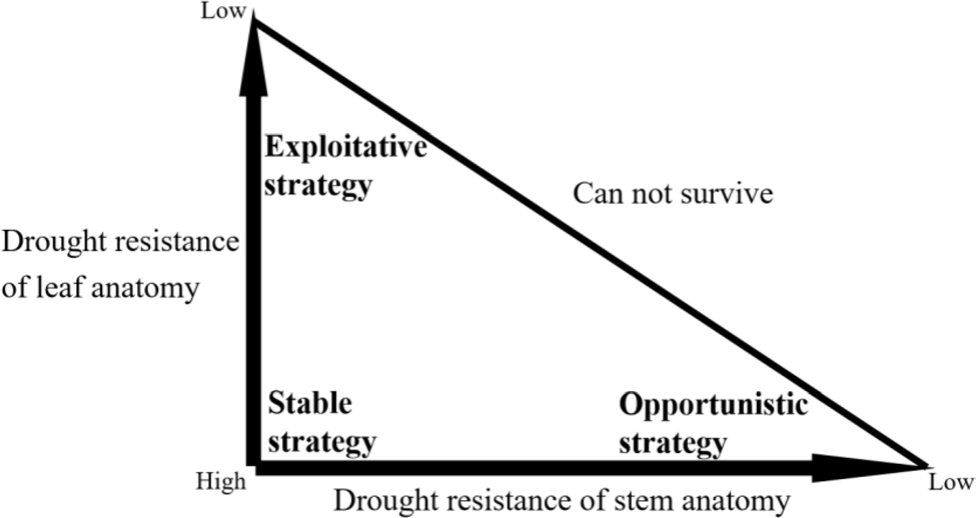
An adaptive strategy triangle based on leaf-stem anatomical traits imitating the CSR strategies

Anatomical traits are comprehensive expressions of plants adaptation to environments [4], and their capacity to explain specific functions is limited. Therefore, we analyze and validate the adaptive strategy at species and community levels by combining morphological and anatomical features (Figure S1). The three adaptive strategies we summarized were consistent with the adaptive characteristics for most desert plants, and basically represent the three main strategies for shrubs adaptation in the eastern Qaidam Basin. Combined those results with habitat information of studied plant species, we established an adaptive strategy system for the anatomical traits of shrub leaf-stem in desert. Three types of strategies were named as exploitative, stable, and opportunistic strategies (Fig. 11).

We defined PC1 as an exploitative strategy, in which stems tended to adopt the safety strategy and leaves tended to adopt the hydraulic efficiency strategy. Leaf with higher La, lower LT and ET increased the contact area with sunlight and air, thus maintaining high physiological activity efficiency [4]. At the same time, the larger single-leaf area and the characteristics of thinner LT, ET and MT reduced the defensive capabilities of the blades. However, as indicated in the ternary graph (Fig. 4), species close to the exploitative strategy were *N. tangutorum* and *A. sphaerocephala*, both with relatively small leaves and branches. Corresponded to the characteristics of exploitative strategy, this might be an effective way for species to reduced transpiration and water loss. Leaves of *N. tangutorum* were typically leathery with an oily surface while *A. sphaerocephala* leaves curled toward the center. These morphological characteristics indicated high drought tolerance. High SA, CT, VD, and low XS pointed to the strategy of high safety and low water transport efficiency, and high VS maintained a certain degree of water transport capacity. In the anatomical images (Figure S1), the pith of *N. tangutorum* and *A. sphaerocephala* was significantly larger than those of other species, which indicated a more drought tolerant stem [4]. Under drought conditions, the medulla swelled composed of large parenchyma cells became the main water storage tissue of the stem to increase the drought tolerance of the stem. Combining stem and leaf strategies, we argue that *N. tangutorum* and *A. sphaerocephala* formed a versatile adaptive strategy with generality for drought-resistant plants. When soil moisture was sufficient, the leaves used hydraulic efficiency strategies to make the plant grow fast. When water was scarce, plants discarded some of the distal “cheap” organs (leaves) to maintain the survival of more “expensive” proximal organs (stems) [4]. *N. tangutorum* and *A. sphaerocephala* mainly habituated in sandy deserts, and the coordinated strategies of stems and leaves effectively utilized all resources and water, occupying a large ecological niche of this habitat through these adaptive strategies.

PC2 was defined as a stable strategy, in which leaves/assimilation branches tended to the hydraulic safety strategy, and the stems tended to the safety strategy, both led to a high level of drought tolerance. Although species- and community-level PCA results indicated different anatomical traits, the overall strategy pointed to a similar finding. At the species level, the thickening of leaf tissues reflected hydraulic safety strategy, and the decrease in stem VS reduced the efficiency of water transport, all of which referred to high drought tolerance characteristics [21–25]. We found that both the total leaf area and the single leaf area contributed higher on the second cardinal axis, but the contribution rate of the total leaf area was higher, which may be the characteristic of small and dense leaves of this strategy, which further improved the protective ability of leaves [4]. The anatomical characteristics of leaves/assimilation branches were different at the species and community levels, but the thickening of leaf tissue and the reduction of duct area were effective ways to reduce transpiration and water retention capacity of leaves, which reflected a high hydraulic security strategy as a whole. There was a high degree of consistency between different levels of stem anatomy in the stability strategy. The thickening of the organ maintained the mechanical strength, and the reduction of the catheter area reduced the efficiency of water transport. These characteristics ensured the safety and water storage of the stem itself, while reduced the upward supply of water to avoid excessive leaf growth and large water loss. The overall performance reflected strong hydraulic safety capabilities. Species that tend to the stable strategy were *K. foliatum* and *H. ammodendron*. The anatomical images (Figure S1) of both species (*K. foliatum* and *H. ammodendron*) showed similar anatomical features of stems and leaves/assimilation branches, with abnormal vascular bundles in xylem greatly improving stem safety [64]. Overall, the safety strategy of leaves combined with the safety strategy of stems led to high drought tolerance and viability for plants. The main habitat of *K. foliatum* and *H. ammodendron* was saline desert (from the Flora of China [65]), and calcium oxalate crystals in mesophyll improved water retention and salinity tolerance [66]. This strategy of stems and leaves/assimilation branches together maintained hydraulic safety (reduced water loss through transpiration and increased water retention capacity) and mechanical strength (the thickening of the organ improved the protective ability, and the reduction of the catheter area reduced the efficiency of water delivery), allowing plants to survive in water-stressed environments.

PC3 could be defined as an opportunistic strategy, as leaves tended toward hydraulic safety strategy and stems tended toward water transport efficiency strategy. Although species- and community-level PCA results indicated different anatomical traits, the overall strategy pointed to a similar finding. The thickening of leaf tissue suggested the shift of leaves to a more drought-tolerant hydraulic security strategy [21–25]. The trend of change in SD and SL was consistent at the species and community level, and the leaves not only improved certain protection ability, but also ensured certain physiological activities. These characteristics largely explain the hydraulic safety strategy of the blades. In opportunistic strategies, stems were more about transported water to the leaves to ensure the normal physiological activities of the leaves. Species-level features (reduced duct density and increased area) demonstrate this strategy. VS enlargement was a rare feature of desert plants, which increased the transpiration and water loss of leaves, which could only be supported by an adequate water supply. Anatomical images of stems and leaves (Figure S1) of opportunistic strategies species (*S. regelii*) showed similar characteristics to those of stable strategy species (*K. foliatum* and *H. ammodendron*), but the number of abnormal vascular bundles in the xylem of *S. regelii* is much lower than that of *K. foliatum* and *H. ammodendron*. Habitats for *S. regelii* included most desert types such as sandy, saline, and gravelly desert. Stem viability of *S. regelii* was low, plants depended on high water transport efficiency to maintain leaf survival. This strategy allowed the species to have a wide range but not occupy a broad niche, waiting for opportunities that are suitable for survival without competition in a variety of habitat conditions and over a large scale.

Both of plant traits and strategies were associated with water use under water stress conditions. Based on our results, the trend of stem and leaf anatomical traits of woody plants in the eastern Qaidam Basin strongly supported the known types of adaptive strategies (e.g., decreased water loss by decreased the number of leaves, the leaf thickening improved the protective capacity, and the density of stem vessel increased to reduce water transport and increased the mechanical support capacity) [4, 67]. Moreover, the commonly used functional traits LMA and WD increased with the increasement in plant with higher drought tolerance, further verifying our view [37, 46]. The adaptive strategy triangle using stem-leaf strategies as the two axes of the coordinate system was in accordance with the three strategies in our results, thus verifying our hypothesis.

### Effects of water stress on leaf-stem anatomical traits and adaptive strategies

The environment of the Qaidam Basin is coldness, drought, and salinization [44]. The characteristics of water stress are consistent with the environmental particularity of the Qaidam Basin [46, 47]. All of the aridity/drought indices used here are indicators that integrate specific environmental factors to evaluate the degree of drought in a region. While SPI can be used to monitor environmental short-term water supply, SPEI is suitable for long-term, large-scale drought assessment [67]. Meanwhile, PDSI is an indicator that provides a comprehensive assessment of the total moisture status of an area [68], and AI indicates atmospheric dryness [69]. The results of the analysis confirmed the environmental characteristics of the Qaidam Basin, and the increase of drought (the decrease of drought index) was mainly caused by the decrease of temperature rather than precipitation, and the low temperature and salinity had a greater impact on the survival and growth of desert plants in the region. Soil conditions were the main controlling factors of water stress in the study area. Compared with dry climate, soil dryness can lead to adaptive variation in the anatomical characteristics of plant stems and leaves.

The environment could comprehensively influence plant growth in nature. The explanation of adaptive strategies by water stress supported the plant adaptive strategy system that we proposed. We found that the exploitative strategy was mainly controlled by salt stress and low-temperature stress, which were the most typical and widely distributed environmental conditions in the Qaidam Basin. The decrease in temperature was accompanied by an increase in precipitation, suggesting that plants might lack the ability to absorb and use water due to frozen temperature. The thickening of leaves and the increase of XS could improve the cold resistance of plants to enable them to cope with coldness stress [4]. Under the premise of having a certain degree of cold tolerance, the stem increased the upward water supply to maintain the physiological activity of the leaves. The intensification of salt stress led to a decrease in leaf protection, but the stem increased viability to ensure plant survival and growth. High pH inhibited plant root activity, thus making it difficult for plants to obtain water [70]. The way in which exploitative strategy responded to both types of stresses implied that there was a trade-off between the anatomical traits of stems and leaves. Plants enhanced physiological activity to achieve balance by tissue thickening to reduce transpiration and water loss [4]. The water supplied to the leaves increased under the condition that the stems ensure their own safety and water storage capacity. Stems and leaves adapted to drought stress through their trade-offs and synergistic relationships. This condition was well suitable for the initiative and stability of plants’ exploitative strategies. The stable strategy was basically controlled by soil factors, and stable strategy plants could form a relatively overall water retention characteristic at both stem and leaf scales. When the severity of drought stress increased, plant stems would improve safety and water retention capacity, thus becoming the main source of water for leaves. Partial leaf shedding reduced transpiration and water loss while ensuring normal physiological activities. When the severity of drought stress decreased, plant would increase their leaf numbers to increase the place of photosynthesis. Stable strategy plants were in a dominant position in the habitat suitable for them. Compared with soil moisture conditions, atmospheric moisture conditions did not have very stable regularity, which was also an important condition for the formation of opportunistic strategies. Plants adopting opportunistic strategy mainly maintained their survival through leaf adaptation, and stems mainly provided sufficient water for leaves [71]. However, as overall safety of opportunistic strategy plants was low, accidental precipitation and temperature changes could change the niche of opportunistic strategies plants, and soil texture was also an important environmental factor affecting the survival of opportunistic strategies plants. However, because of this, opportunistic strategies plants had the potential to gain a larger growth range, and more communities might appear in them. Overall, plants stabilized in various habitats through different adaptive strategies [72, 73].

The anatomical structure of the plant was a direct fa ctor that determines the function of the plant, and the anatomy responded to the harsh habitat conditions through a certain degree of adaptive variation. Leaves were organs that underwent more adaptive variation and were more likely to be “sacrificed” than more valuable organs (stems). We found that LA, LT, ET of leaves and XS, VD and VS of stems were more adaptive anatomical traits regardless of the adaptive strategy. But at the same time, we were surprised that the contribution of leaf stomata to adaptive strategies, contributed only in the opportunistic strategies, which was theoretically important for water retention and water loss, was not obvious. This might be due to that the stomata had developed a more stable strategy and were less affected by the environment. It could also be due to the small scope of our research and database, which is something we need to improve later. Overall, the relationship between the environment and the three adaptive strategies is a good validation of our hypothesis, and the adaptation strategy system based on stem and leaf anatomical traits designed at the species and community levels can be explained by the environment.

## Conclusions

The adaptive strategies of desert shrubs are results of the synthesis of variations of multiple anatomical traits. Water stress also occurs due to a combination of environmental factors. Evidence of anatomical variations could support existing adaptive strategy types. By combining current adaptative strategy theory and environmental influences of anatomical traits at the species and community levels, we established an adaptive strategy system based on leaf-stem anatomical traits for desert shrubs. After the verification of water stress, we identified three dimensions of exploitative, stable, and opportunistic strategy in the system. With more refinement and validation on our system in the future, a more mature adaptive strategy system based on the anatomical traits of desert plants will be established.

### Electronic supplementary material

Below is the link to the electronic supplementary material.

Supplementary Material 1

## Data Availability

All trait data are collected and experimented by the authors, and there is no conflict of interest.All plant data are measured and publicly available and uploaded with attachments. Meteorological data from the National Earth System Science Data Center: http://www.geodata.cn/data/index.html?publisherGuid=126744287495931&categoryId=4.
