# Supplementary material for: Adaptive strategies based on shrub leaf-stem anatomy and their environmental interpretations in the eastern Qaidam Basin
Source: BMC Plant Biol. 2024 Apr 24;24:323. doi: 10.1186/s12870-024-05026-3 (PMC11040798; doi:10.1186/s12870-024-05026-3)
Supplement: Supplementary file 1 — Supplementary Material 1 [file 12870_2024_5026_MOESM1_ESM.docx]

Table S1 Sample site location information

| **Site** | **Geographic position** | **Altitude (m)** | **[area](javascript:;)** |
| --- | --- | --- | --- |
| **P01** | 36.16°N, 97.19°E | 2892 | Duran County |
| **P02** | 36.05°N, 97.45°E | 2970 | Duran County |
| **P03** | 36.50°N, 98.04°E | 3066 | Duran County |
| **P04** | 36.45°N, 98.06°E | 3070 | Duran County |
| **P05** | 36.48°N, 98.33°E | 3304 | Duran County |
| **P06** | 36.66°N, 98.05°E | 3120 | Duran County |
| **P07** | 37.11°N, 97.51°E | 2857 | Delingha City |
| **P08** | 37.06°N, 97.60°E | 2858 | Delingha City |
| **P09** | 37.04°N, 97.83°E | 2917 | Delingha City |
| **P10** | 36.79°N, 99.03°E | 3048 | Ulan County |
| **P11** | 36.81°N, 98.64°E | 3014 | Ulan County |
| **P12** | 37.33°N, 98.07°E | 3402 | Delingha City |

Table S2 Detailed species information

| **Family** | **Species** | **Life form** | **Leaf texture** | **[Habitat](http://dict.cn/The%20plant%20habitat)** |
| --- | --- | --- | --- | --- |
| Nitrariaceae | Nitraria tangutorum | shrub | leathery | sandy desert |
| Asteraceae | Artemisia sphaerocephala | subshrub | leathery | sandy, saline desert |
| Amaranthaceae | Sympegma regelii | dwarf shrub | fleshy | gravelly, sandy, saline desert |
| Amaranthaceae | Haloxylon ammodendron | small tree or shrub | fleshy | sandy, saline desert |
| Amaranthaceae | Kalidium foliatum | dwarf shrub | fleshy | saline desert |

Table S3 Specific data on species anatomical traits

|  | ***N. tangutorum*** | ***A. sphaerocephala*** | ***S. regelii*** | ***H. ammodendron*** | ***K. foliatum*** |
| --- | --- | --- | --- | --- | --- |
| **LMA** | 0.953±0.053a | 0.302±0.020a | 0.244±0.053a | 0.768±0.055a | 0.247±0.100a |
| **LA** | 68.967±28.441a | 18.757±0.041a | 31.494±5.362a | 47.801±1.036a | 85.459±19.928a |
| **La** | 0.377±0.122a | 0.065±0.018c | 0.208±0.040bc | 0.269±0.024ab | 0.101±0.056c |
| **LT** | 1058.088±73.786a | 988.644±53.854a | 1829.841±25.158b | 2164.215±182.658b | 2100.974±136.491b |
| **ET** | 56.149±0.097c | 36.096±3.684c | 89.686±6.822bc | 111.835±33.401b | 193.733±14.165a |
| **MT** | 203.191±12.906c | 384.142±54.715b | 668.910±189.214a | 938.764±33.822a | 762.144±192.469a |
| **SD** | 11.861±0.236a | 7.389±0.039a | 10.722±0.904a | 11.852±0.393a | 8.589±0.891a |
| **SL** | 9.232±0.637a | 12.158±2.065a | 15.031±0.798a | 11.211±0.219a | 10.696±1.034a |
| **WD** | 0.753±0.053a | 0.673±0.036b | 0.676±0.033b | 0.726±0.026ab | 0.661±0.041b |
| **SA** | 0.103±0.007a | 0.037±0.014b | 0.042±0.007b | 0.106±0.036a | 0.039±0.012b |
| **CT** | 0.064±0.012a | 0.048±0.007ab | 0.061±0.018a | 0.043±0.005ab | 0.036±0.013b |
| **XS** | 0.156±0.025b | 0.156±0.049b | 0.307±0.017a | 0.231±0.017ab | 0.291±0.071ab |
| **PS** | 0.253±0.030ab | 0.318±0.062a | 0.193±0.026ab | 0.145±0.052b | 0.206±0.140ab |
| **VD** | 100.500±7.425a | 88.500±7.263a | 57.833±11.251b | 66.333±6.364b | 62.400±7.024b |
| **VS** | 30.716±2.679a | 34.399±0.529a | 28.261±4.988ab | 26.265±0.728ab | 19.592±1.784b |

Table S4 Specific data on anatomical traits of community leaves

| **Site** | **LMA** | **LA** | **La** | **LT** | **ET** | **MT** | **SD** | **SL** |
| --- | --- | --- | --- | --- | --- | --- | --- | --- |
| **P01** | 0.693±0.002cd | 66.109±25.093ab | 0.238±0.004c | 1829.292±185.921abc | 86.714±7.745b | 754.925±47.264b | 9.738±4.532abc | 13.288±0.236bc |
| **P02** | 0.219±0.008cd | 27.528±2.437bc | 0.142±0.001g | 1663.406±1.321bcd | 86.707±1.686b | 663.982±70.670b | 10.938±1.000abc | 13.709±1.749a |
| **P03** | 0.336±0.011ab | 10.487±1.741c | 0.185±0.005e | 1133.568±28.989e | 39.227±5.454b | 475.122±38.185d | 14.511±1.890ab | 10.634±2.218cd |
| **P04** | 0.112±0.009bcd | 6.944±1.613c | 0.049±0.001j | 906.791±57.020de | 34.802±4.767b | 433.127±2.205c | 7.932±0.501cd | 15.367±1.033ab |
| **P05** | 0.209±0.000d | 70.231±6.317ab | 0.112±0.001h | 2284.706±23.783ab | 185.580±19.994a | 762.144±69.065b | 7.889±1.834cd | 12.363±1.636bcd |
| **P06** | 0.141±0.000d | 68.020±18.937ab | 0.104±0.004h | 1725.036±55.742abc | 182.261±1.910a | 655.182±112.979b | 7.403±1.072cd | 8.257±0.863cd |
| **P07** | 0.393±0.023a | 18.890±1.825bc | 0.228±0.005d | 1179.029±0.989cde | 63.061±1.411b | 543.514±21.906c | 7.389±0.629cd | 7.984±0.387cd |
| **P08** | 1.487±0.016abc | 94.984±33.258ab | 0.306±0.006a | 1384.462±23.303abc | 75.763±1.326b | 602.385±12.879c | 9.958±0.000abc | 7.966±1.075d |
| **P09** | 0.412±0.000d | 105.720±44.265a | 0.060±0.002i | 1611.014±46.618a | 130.606±12.372a | 1057.503±81.700a | 6.556±0.679d | 10.383±1.341cd |
| **P10** | 0.233±0.000d | 95.116±27.855a | 0.030±0.003k | 1859.792±10.955abc | 172.733±1.752b | 773.593±70.278b | 8.576±1.667bcd | 8.309±0.189cd |
| **P11** | 0.287±0.000d | 36.319±9.138bc | 0.240±0.005b | 1974.426±43.364ab | 67.942±4.240b | 1035.977±86.015b | 13.500±1.000a | 17.026±1.532ab |
| **P12** | 0.181±0.001d | 63.363±27.671abc | 0.173±0.003f | 2449.265±54.549a | 240.087±6.679b | 1180.415±201.874a | 9.986±0.833abc | 11.497±0.268cd |

Table S5 Specific data on community stem anatomical traits

| **Site** | **WD** | **SA** | **CT** | **XS** | **PS** | **VD** | **VS** |
| --- | --- | --- | --- | --- | --- | --- | --- |
| **P01** | 0.649±0.037bc | 0.090±0.004ab | 0.052±0.009a | 0.252±0.014b | 0.153±0.034b | 57.560±1.414c | 23.553±2.849cde |
| **P02** | 0.652±0.091bc | 0.044±0.059a | 0.070±0.031a | 0.266±0.040b | 0.240±0.133ab | 54.112±2.828bc | 31.579±0.929ab |
| **P03** | 0.697±0.039bcd | 0.065±0.022cd | 0.050±0.015abc | 0.176±0.007b | 0.243±0.071ab | 87.562±0.707abc | 36.554±4.118ab |
| **P04** | 0.605±0.019bc | 0.024±0.006d | 0.053±0.017ab | 0.135±0.036b | 0.278±0.037a | 76.553±19.502ab | 32.281±2.419abc |
| **P05** | 0.687±0.032cd | 0.028±0.009d | 0.057±0.005bc | 0.400±0.040b | 0.447±0.066a | 52.000±6.245c | 30.897±3.030e |
| **P06** | 0.511±0.025abc | 0.027±0.003d | 0.025±0.007bc | 0.272±0.103ab | 0.179±0.014ab | 58.087±11.314abc | 16.300±3.858de |
| **P07** | 0.723±0.019bc | 0.066±0.017abc | 0.049±0.010ab | 0.172±0.092b | 0.249±0.064ab | 91.247±2.828abc | 32.356±4.820bc |
| **P08** | 0.760±0.024ab | 0.120±0.025d | 0.065±0.010abc | 0.125±0.034b | 0.211±0.111ab | 95.773±21.213a | 23.172±2.168bcd |
| **P09** | 0.654±0.026a | 0.041±0.003d | 0.028±0.007c | 0.216±0.047ab | 0.151±0.014ab | 60.000±7.937bc | 16.063±1.218e |
| **P10** | 0.585±0.029bcd | 0.034±0.003d | 0.023±0.007c | 0.236±0.027ab | 0.093±0.051ab | 47.909±2.121bc | 11.266±1.893de |
| **P11** | 0.700±0.038bcd | 0.036±0.011bcd | 0.045±0.008abc | 0.298±0.044ab | 0.197±0.077ab | 70.500±7.095bc | 29.308±2.540abc |
| **P12** | 0.695±0.074e | 0.057±0.016d | 0.040±0.002abc | 0.250±0.088b | 0.117±0.077ab | 76.618±6.110abc | 18.877±0.601de |

Table S6 Environmental data indicators within the sample site

| **Variable** | P01 | P02 | P03 | P04 | P05 | P06 | P07 | P08 | P09 | P10 | P11 | P12 |
| --- | --- | --- | --- | --- | --- | --- | --- | --- | --- | --- | --- | --- |
| SPI | -1.000 | 0.270 | -1.130 | -1.060 | -0.830 | -1.020 | -0.320 | -0.600 | -0.740 | -0.810 | -0.770 | -0.040 |
| SPEI | 1.393 | 1.356 | 1.747 | 1.747 | 1.793 | 1.883 | 1.924 | 1.932 | 1.944 | 1.868 | 1.934 | 1.993 |
| PDSI | 2.800 | 3.300 | 4.600 | 4.700 | 3.800 | 4.700 | 4.700 | 4.600 | 5.000 | 3.700 | 2.700 | 2.600 |
| AI | 5.234 | 3.341 | 5.685 | 5.389 | 4.296 | 5.354 | 5.992 | 6.161 | 5.765 | 4.139 | 4.582 | 3.897 |
| pH | 8.722 | 9.374 | 9.260 | 9.287 | 8.639 | 8.571 | 9.684 | 9.570 | 8.429 | 9.143 | 9.549 | 8.911 |
| EC | 11.332 | 9.364 | 9.537 | 9.593 | 11.157 | 11.227 | 9.239 | 9.993 | 15.777 | 10.217 | 9.336 | 10.640 |
| WS | 1.877 | 1.153 | 0.957 | 3.679 | 6.718 | 3.474 | 0.827 | 5.686 | 8.532 | 9.307 | 0.624 | 6.660 |
| SH | 20.301 | 25.426 | 28.196 | 23.257 | 34.719 | 39.179 | 42.956 | 39.379 | 62.089 | 43.513 | 32.474 | 38.670 |
| BD | 1.308 | 1.397 | 1.526 | 1.525 | 1.346 | 1.273 | 1.523 | 1.501 | 1.312 | 1.288 | 1.375 | 1.239 |
| SOM | 1.304 | 0.740 | 0.851 | 0.856 | 1.937 | 3.685 | 0.710 | 1.682 | 9.092 | 6.430 | 4.264 | 5.259 |
| MAP | 187.996 | 285.545 | 165.617 | 171.339 | 191.870 | 167.908 | 158.240 | 156.181 | 159.451 | 203.618 | 191.646 | 183.923 |
| MPS | 34.083 | 54.873 | 27.877 | 29.052 | 33.124 | 35.926 | 29.445 | 28.322 | 28.607 | 36.589 | 33.307 | 36.095 |
| AP8 | 26.365 | 41.687 | 21.542 | 22.720 | 28.267 | 28.603 | 22.485 | 21.264 | 21.165 | 33.296 | 28.623 | 24.564 |
| MAE | 983.900 | 953.900 | 941.500 | 923.300 | 824.300 | 899.000 | 948.100 | 962.200 | 919.200 | 842.700 | 878.200 | 716.800 |
| MAH | 27.302 | 32.606 | 35.080 | 38.030 | 41.170 | 38.110 | 46.519 | 35.969 | 37.181 | 43.709 | 41.891 | 49.445 |
| MAT | 1.532 | -1.340 | 4.882 | 4.529 | 1.909 | 4.459 | 5.296 | 4.916 | 3.944 | 1.029 | 0.791 | 0.809 |
| MTS | 12.555 | 9.229 | 16.220 | 15.801 | 12.971 | 10.715 | 17.674 | 17.128 | 15.833 | 11.803 | 11.867 | 12.569 |
| AT8 | 13.541 | 10.212 | 16.952 | 16.516 | 13.638 | 11.349 | 18.368 | 17.846 | 16.602 | 12.546 | 12.653 | 13.320 |
| MAW | 2.157 | 1.716 | 2.708 | 2.347 | 2.010 | 2.326 | 1.659 | 2.504 | 2.525 | 2.070 | 1.412 | 1.763 |

Table S7 Explanatory rates and eigenvalues for species and community principal component analysis

| **Species** | **Eigenvalue** | **Percentage of variance (％)** | **Cumulative**  **(％)** | **Community** | **Eigenvalue** | **Percentage of variance (％)** | **Cumulative**  **(％)** |
| --- | --- | --- | --- | --- | --- | --- | --- |
| Dim1 | 5.407 | 0.360 | 0.360 | Dim1 | 5.668 | 0.378 | 0.378 |
| Dim2 | 3.344 | 0.223 | 0.583 | Dim2 | 3.336 | 0.222 | 0.600 |
| Dim3 | 1.864 | 0.124 | 0.708 | Dim3 | 2.507 | 0.167 | 0.767 |
| Dim4 | 1.333 | 0.089 | 0.797 | Dim4 | 1.372 | 0.091 | 0.859 |
| Dim5 | 0.987 | 0.066 | 0.862 | Dim5 | 0.746 | 0.050 | 0.909 |
| Dim6 | 0.559 | 0.037 | 0.900 | Dim6 | 0.573 | 0.038 | 0.947 |
| Dim7 | 0.506 | 0.034 | 0.933 | Dim7 | 0.309 | 0.021 | 0.967 |
| Dim8 | 0.372 | 0.025 | 0.958 | Dim8 | 0.250 | 0.017 | 0.984 |
| Dim9 | 0.279 | 0.019 | 0.977 | Dim9 | 0.161 | 0.011 | 0.995 |
| Dim10 | 0.197 | 0.013 | 0.990 | Dim10 | 0.050 | 0.003 | 0.998 |
| Dim11 | 0.098 | 0.007 | 0.996 | Dim11 | 0.027 | 0.002 | 1.000 |
| Dim12 | 0.035 | 0.002 | 0.999 | Dim12 | 0.000 | 0.000 | 1.000 |
| Dim13 | 0.013 | 0.001 | 0.999 | Dim13 | 0.000 | 0.000 | 1.000 |
| Dim14 | 0.007 | 0.000 | 1.000 | Dim14 | 0.000 | 0.000 | 1.000 |
| Dim15 | 0.001 | 0.000 | 1.000 | Dim15 | 0.000 | 0.000 | 1.000 |

Table S8 Trait dimensionality reduction values for species and community principal component analysis

| **Species** | Dim1 | Dim2 | Dim3 | **Community** | Dim1 | Dim2 | Dim3 |
| --- | --- | --- | --- | --- | --- | --- | --- |
| LMA | 0.261 | 0.313 | 0.008 | LMA | 0.218 | 0.411 | 0.056 |
| LA | 0.001 | 0.367 | -0.146 | LA | -0.237 | 0.383 | 0.060 |
| La | 0.243 | 0.223 | 0.244 | La | 0.284 | 0.231 | 0.295 |
| LT | -0.296 | 0.297 | 0.242 | LT | -0.270 | 0.071 | 0.462 |
| ET | -0.327 | 0.214 | -0.066 | ET | -0.345 | 0.125 | 0.160 |
| MT | -0.335 | 0.222 | 0.112 | MT | -0.244 | 0.152 | 0.367 |
| SD | 0.107 | 0.218 | 0.491 | SD | 0.197 | -0.073 | 0.277 |
| SL | -0.113 | -0.170 | 0.505 | SL | 0.045 | -0.344 | 0.288 |
| WD | 0.303 | 0.245 | 0.105 | WD | 0.275 | 0.160 | 0.331 |
| SA | 0.271 | 0.378 | 0.066 | SA | 0.257 | 0.397 | 0.093 |
| CT | 0.272 | -0.057 | 0.321 | CT | 0.313 | -0.106 | 0.235 |
| XS | -0.312 | -0.074 | 0.295 | XS | -0.239 | -0.192 | 0.404 |
| PS | 0.127 | -0.355 | 0.066 | PS | 0.132 | -0.314 | 0.133 |
| VD | 0.363 | -0.006 | -0.246 | VD | 0.331 | 0.158 | -0.077 |
| VS | 0.240 | -0.346 | 0.277 | VS | 0.317 | -0.329 | 0.113 |

Table S9 Dimensionality reduction results in principal component analysis of traits

| **Species** | **Eigenvalue** | **Percentage of variance (％)** | **Cumulative**  **(％)** |
| --- | --- | --- | --- |
| PC1 | 0.428 | 1.029 | 0.895 |
| PC2 | 0.715 | -1.900 | 0.769 |
| PC3 | 3.247 | -0.987 | -0.566 |
| PC4 | 1.479 | -2.871 | -2.449 |
| PC5 | -1.478 | -2.009 | 2.270 |
| PC6 | -3.059 | -0.051 | -1.781 |
| PC7 | 2.528 | 0.216 | -1.242 |
| PC8 | 3.685 | 4.016 | 0.142 |
| PC9 | -2.461 | 1.306 | -0.848 |
| PC10 | -3.601 | 1.013 | -1.508 |
| PC11 | 0.474 | -1.141 | 2.461 |
| PC12 | -1.956 | 1.380 | 1.857 |


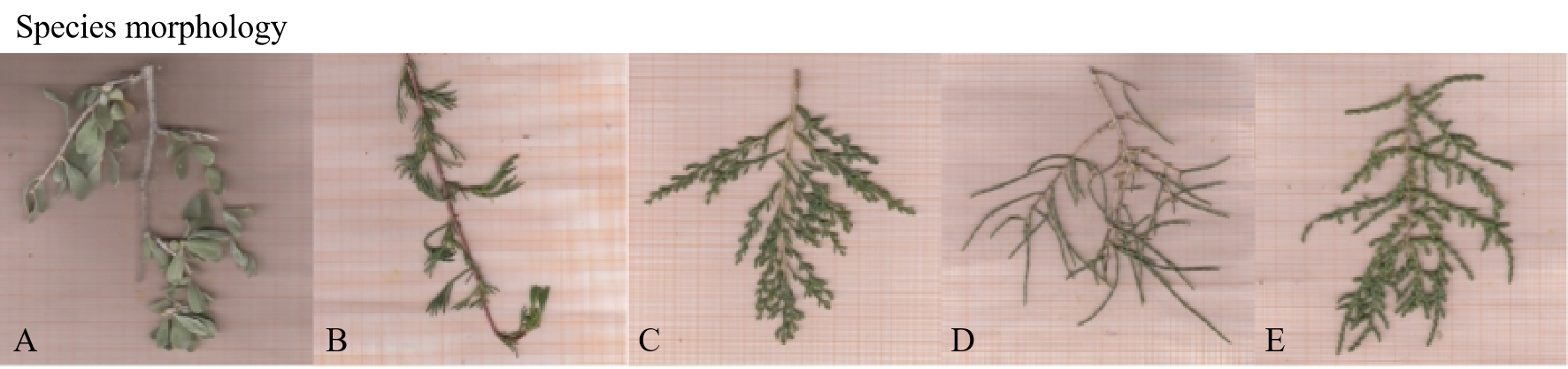

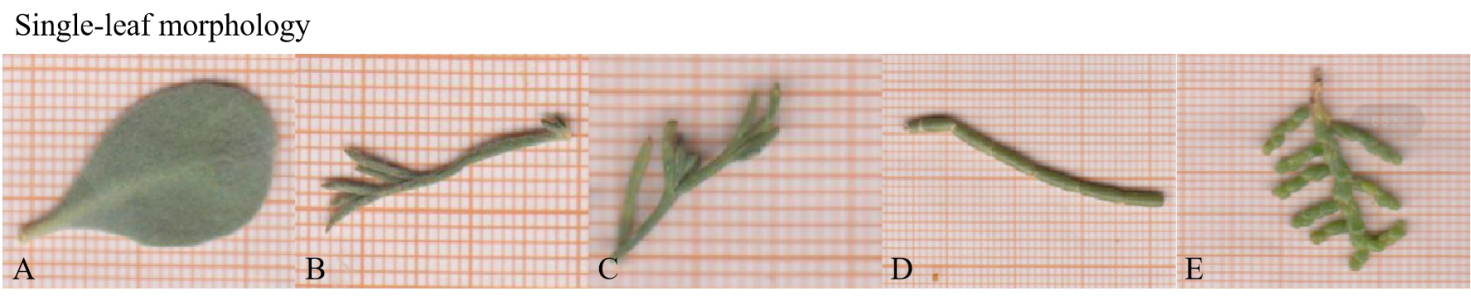

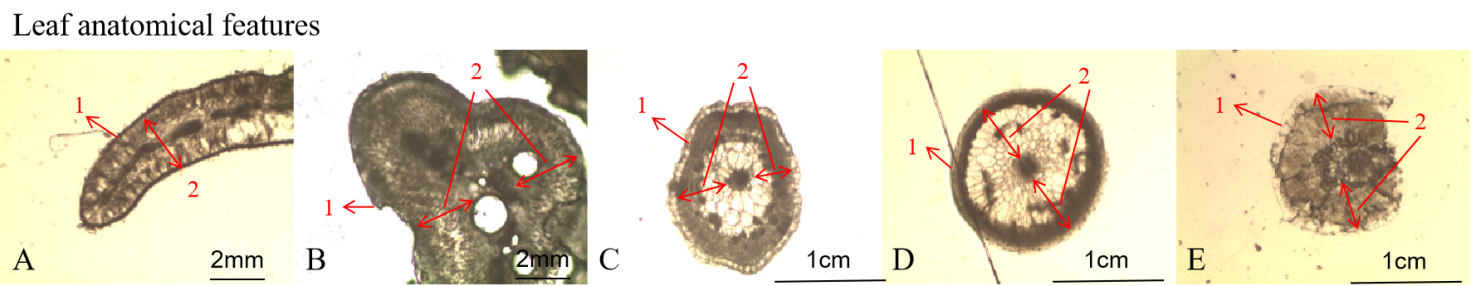

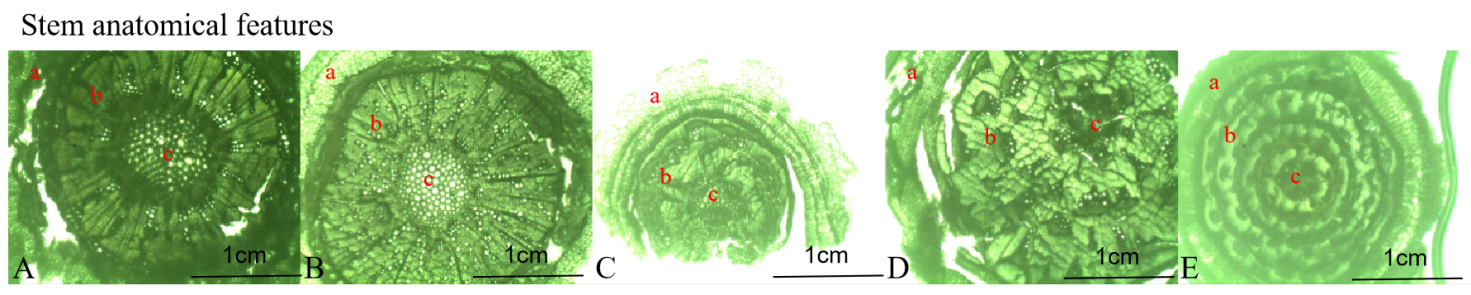


Figure S1 Morphological and anatomical images of five species. From top to bottom, species morphology, single leaf morphology, leaf anatomical traits, and stem anatomical traits are in order. A: *N. tangutorum*; B: *A. sphaerocephala*; C: *S. regelii*; D: *H. ammodendron*; E: *K. foliatum*. In leaf anatomy images: 1: Leaf epidermis; 2: Mesophyll. In stem anatomy images: a: Stem cortex; b: Xylem; c: Pith. The lower right corner of the picture is a scale bar.
